# Real‐time radial reconstruction with domain transform manifold learning for MRI‐guided radiotherapy

**DOI:** 10.1002/mp.16224

**Published:** 2023-01-27

**Authors:** David E. J. Waddington, Nicholas Hindley, Neha Koonjoo, Christopher Chiu, Tess Reynolds, Paul Z. Y. Liu, Bo Zhu, Danyal Bhutto, Chiara Paganelli, Paul J. Keall, Matthew S. Rosen

**Affiliations:** ^1^ Image X Institute, Faculty of Medicine and Health The University of Sydney Sydney Australia; ^2^ Department of Medical Physics Ingham Institute for Applied Medical Research Liverpool NSW Australia; ^3^ A. A. Martinos Center for Biomedical Imaging Massachusetts General Hospital Charlestown Massachusetts USA; ^4^ Department of Biomedical Engineering Boston University Boston Massachusetts USA; ^5^ Dipartimento di Elettronica, Informazione e Bioingegneria Politecnico di Milano Milan Italy; ^6^ Department of Physics Harvard University Cambridge Massachusetts USA; ^7^ Harvard Medical School Boston Massachusetts USA

**Keywords:** deep learning, MRI, radiotherapy

## Abstract

**Background:**

MRI‐guidance techniques that dynamically adapt radiation beams to follow tumor motion in real time will lead to more accurate cancer treatments and reduced collateral healthy tissue damage. The gold‐standard for reconstruction of undersampled MR data is compressed sensing (CS) which is computationally slow and limits the rate that images can be available for real‐time adaptation.

**Purpose:**

Once trained, neural networks can be used to accurately reconstruct raw MRI data with minimal latency. Here, we test the suitability of deep‐learning‐based image reconstruction for real‐time tracking applications on MRI‐Linacs.

**Methods:**

We use automated transform by manifold approximation (AUTOMAP), a generalized framework that maps raw MR signal to the target image domain, to rapidly reconstruct images from undersampled radial k‐space data. The AUTOMAP neural network was trained to reconstruct images from a golden‐angle radial acquisition, a benchmark for motion‐sensitive imaging, on lung cancer patient data and generic images from ImageNet. Model training was subsequently augmented with motion‐encoded k‐space data derived from videos in the YouTube‐8M dataset to encourage motion robust reconstruction.

**Results:**

AUTOMAP models fine‐tuned on retrospectively acquired lung cancer patient data reconstructed radial k‐space with equivalent accuracy to CS but with much shorter processing times. Validation of motion‐trained models with a virtual dynamic lung tumor phantom showed that the generalized motion properties learned from YouTube lead to improved target tracking accuracy.

**Conclusion:**

AUTOMAP can achieve real‐time, accurate reconstruction of radial data. These findings imply that neural‐network‐based reconstruction is potentially superior to alternative approaches for real‐time image guidance applications.

## 1 INTRODUCTION

Image‐guided radiotherapy is a pillar of modern cancer treatment as it enables the noninvasive treatment of tumors with millimeter‐scale accuracy while causing minimal damage to surrounding healthy tissue.^1^ At the cutting‐edge of radiation oncology is a treatment machine known as an MRI‐Linac, which combines the unrivaled image quality of magnetic resonance imaging (MRI) with linear accelerator (Linac) x‐ray radiation therapy.^2^ Commercial MRI‐Linacs are already achieving new standards of precision radiotherapy through image‐guided adaptation to daily anatomical changes,^3^ with cutting‐edge developments including the implementation of gating techniques that dynamically shutter the radiation beam to account for patient motion.^4^ The next generation of MRI‐Linac technology promises to track tumor motion with a moving radiation beam on the basis of real‐time MRI.^5^ However, the accuracy of these targeting approaches, which are likely to improve patient outcomes and reduce side effects,^6^ is limited by the low spatio‐temporal resolution of MRI.^7^

Fast MRI acquisitions based on acquiring raw k‐space data with sparsely sampled golden‐angle radial trajectories have shown much promise for tumor tracking during MRI‐Linac treatments. These radial trajectories are unique in enabling reconstruction of high‐spatial‐resolution, motion‐robust images^8^ in parallel with high‐temporal‐resolution images from the same raw data.^9^ Implementation of such imaging strategies would be advantageous in the radiotherapy context where low latency imaging is often reluctantly prioritized over resolution.^10^ However, gold‐standard techniques for analytic reconstruction of such undersampled MRI data are computationally slow, presenting a barrier to the real‐time imaging required for dynamic treatment adaptation.^11^, ^12^

**FIGURE 1:**
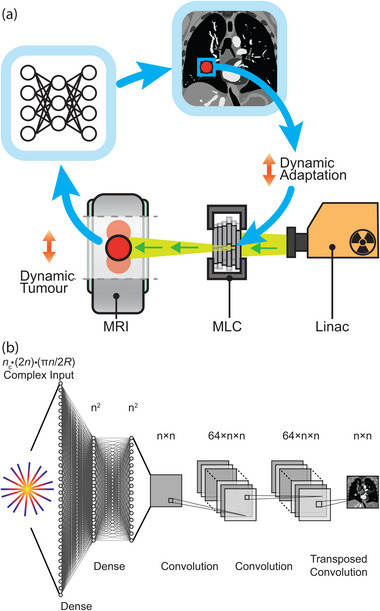
Deep neural networks as a fast, accurate reconstruction technique for tumor tracking applications. (a) Workflow showing the potential role of AUTOMAP in a radiotherapy treatment with dynamic beam adaptation. Dynamic MRI scans are acquired on an MRI‐linac and reconstructed in real time with AUTOMAP. A template‐matching algorithm extracts the target position from images and dynamically adapts the X‐ray beam via a multi‐leaf collimator (MLC). (b) The deep neural network architecture implemented to reconstruct an n×n image from radially sampled MRI data with AUTOMAP. Radial k‐space data are flattened into a 1D vector to create the input to a series of dense and convolutional layers that reconstruct an image.

Deep neural networks have fueled recent progress in computer vision, leading to new technologies across diverse fields such as autonomous vehicles,^13^ molecular analysis,^14^ and medical imaging.^15^, ^16^ Common to many of these technologies is the requirement for pipelines that convert information acquired in an abstract sensor domain to an interpretable format on which real‐world actions can be based. Recently, neural networks have enabled fast, accurate reconstruction of undersampled MRI data.^17^, ^18^ Frameworks that directly reconstruct target images from the raw MRI signal are of particular interest for real‐time applications.^19^, ^20^, ^21^ However, despite these prospects, the successful deployment of neural networks for real‐time imaging applications on systems including MRI‐Linacs (see Fig 1a) still hinges on the availability of training data and utilization of a reconstruction framework suitable for more challenging, nonuniformly sampled image reconstruction.^22^, ^23^, ^24^, ^25^ In particular, there is a dearth of training data for acquisitions corrupted by nonrigid motion, as a static ground truth does not exist.^26^, ^27^, ^28^

One approach to performing real‐time radial reconstruction is the use of automated transform by manifold approximation (AUTOMAP), a generalized neural‐network reconstruction framework that learns the transformation from the raw MR signal to the target image domain from a training corpus built using the forward‐encoding model.^19^ Once trained, this machine‐learning‐based framework reconstructs images in a single forward pass.

Here, we train AUTOMAP to reconstruct undersampled golden‐angle radial trajectory MR data. Using retrospectively acquired data from lung cancer patients, we compare the performance of AUTOMAP to conventional iterative methods for compressed sensing (CS) reconstruction, showing AUTOMAP gives similar reconstruction accuracy but with much faster processing times. Further, we leverage the YouTube‐8M database to synthesize radial k‐space data acquired in the presence of generic motion but with a known ground truth. We then show that our motion‐trained AUTOMAP model leads to more accurate tumor targeting in a digital lung cancer phantom.^29^ These results will guide the development of neural network reconstruction techniques for low‐latency, high accuracy reconstruction in real‐time adaptive radiotherapy.

## 2 METHODS

### 2.1 Model architecture and training hyperparameters

We implemented AUTOMAP using the architecture shown in Figure 1b with Keras (2.4.3) operating on a TensorFlow backend (2.5.0).^19^ The AUTOMAP architecture was created with five trainable layers. The first two layers are dense with hyperbolic tangent activations and map flattened input data through hidden $n^{2}\times 1$ layers. Data are reshaped to an n×n matrix. Data then pass through two convolutional layers with 64 filters, kernel size 5 × 5, and rectified linear activation functions before a transposed convolution with one filter and a 7 × 7 kernel produces the final n×n image. Models were trained to reconstruct images with n=128, with a different model for each acceleration factor.

Training utilized the Adaptive Moment Estimation (Adam) optimizer with a learning rate of 10^−5^, a batch size of 20, and a mean square error (MSE) loss function. Model weights corresponding to the minimum validation cost achieved in 300 epochs of training, with a patience of 15, were saved for reconstruction. For reconstruction of lung cancer patient images, models trained on ImageNet were fine‐tuned for up to 100 epochs with a learning rate of 10^−6^. All computation utilized a NVIDIA RTX 8000 or NVIDIA A6000 graphical processing unit (GPU) on an Ubuntu 18.04 workstation with 128 GB RAM and a 10‐core 3.5 GHz Intel central processing unit (CPU).

### 2.2 Image data preprocessing

We begin by training AUTOMAP to perform image reconstruction under the assumption that anatomy is static. For initial model training, datasets of 20 000 training images and 1000 validation images depicting generic objects were sourced from ImageNet and augmented four times via a series of flips and rotations.^30^ Such large datasets are beneficial to the data‐driven AUTOMAP training process and are easily obtained from ImageNET. We note that as dataset sizes increase, model performance benefits from an increase in the train/test split ratio.^31^ ImageNet data were converted to normalized, grayscale images at 256× 256 resolution and augmented further via addition ofsynthetic phase maps. These synthetic phase maps consist of smoothly varying, two‐dimensional sinusoidal waves with randomly generated frequency and phase offsets. Examples of these phase maps are provided in Figure S1. We highlight that data augmentation with these phase maps, which are fully described in Ref. 19, prevents AUTOMAP from overfitting during training.

Using the MATLAB toolbox for realistic analytical phantoms described in Ref. 32, the ImageNet derived datasets were encoded with golden‐angle radial trajectories to generate single channel ($n_{c}=1$) k‐space datasets for 128× 128 resolution reconstruction via a nonuniform fast Fourier transform (NUFFT) operation. As is standard on clinical MRI scanners, golden‐angle trajectories were oversampled two times in the frequency‐encode direction, which yielded 256 complex data points for each readout spoke. Data were undersampled in the phase‐encode direction by reduction factors (*R*) of 1, 2, 4, 8, and 16, which corresponded to radial trajectories with 202, 101, 51, 26, and 13 spokes, respectively.

After preprocessing, the number of complex datapoints at the flattened AUTOMAP input is

(1) $$n_{c}\cdot \left(2n\right)\cdot \left(\pi n/2R\right),$$

where $n_{c}$ is the number of channels, n=128 is the fully sampled n×n image resolution and *R* is the reduction factor applied in the phase encode direction. The 2*n* term represents 2 × oversampling in readouts and nπ/2R reflects the number of radial views at a reduction factor of *R*. Complex data are separated into real and imaginary components that are concatenated into the flattened input to the first dense layer.

The 256 × 256 grayscale images were downsampled via bilinear interpolation to 128 × 128 for use as ground truth target images in model training.

For model fine‐tuning and evaluation, MRI scans from a 13 patient lung cancer dataset were split in the ratio 9/2/2 (training/validation/testing). This lung cancer dataset is fully described in Refs. 33, 34. Radial, single‐coil k‐space data for these images were retrospectively acquired from images saved in the Digital Imaging and Communications in Medicine (DICOM) format with the procedure described above. Slices from T1‐weighted, T2‐weighted, and cine‐MRI scans were analyzed individually, yielding several hundred images per patient. To simulate real‐world sensor noise, additional k‐space datasets with 25 dB of additive white Gaussian noise (AWGN) was added created with the Signal Processing Toolbox in MATLAB.

### 2.3 Motion data preprocessing

The next part of work this aims to account for intra‐acquisition motion in the neural network reconstruction by incorporating generic motion into the training corpus. Here, we aim to make the motion‐correction generalize by using videos from YouTube as a source of motion‐encoded training data.

For motion training, 7856 image sequences were extracted from 767 videos in the “wildlife” class of the YouTube‐8M database and split randomly into training/validation sets at the ratio 0.85/0.15.^35^ The extraction process ensured that the video sequences contained continuous, smooth motion by excluding cases where the structural similarity (SSIM) between any adjacent frames was lower than 0.94. Sequences where the SSIM between first and last frames was lower than 0.5 were also excluded. These thresholds were chosen as typical of the SSIM values observed across the cine‐MRI lung data described above.

**FIGURE 2:**
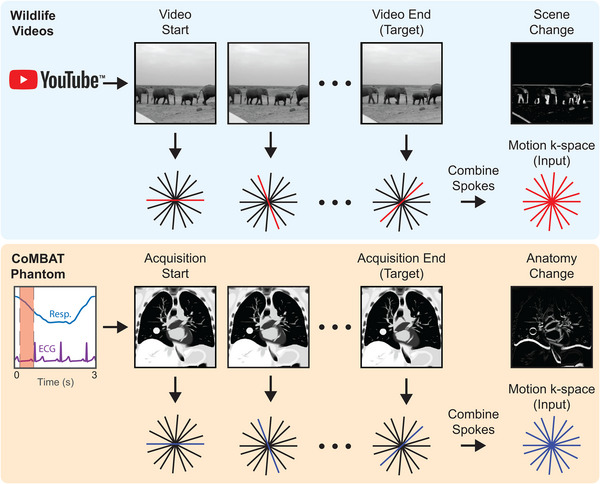
Simulating patient motion during radial acquisitions. The CoMBAT phantom inputs respiratory and ECG traces to simulate patient anatomy during cardiothoracic motion. MR slices are simulated at each timepoint during the acquisition (red shading) and encoded to a golden‐angle radial trajectory. A “motion‐encoded” k‐space is derived by taking individual spokes from the “static” k‐space at individual timepoints during the acquisition. The anatomy change between start and end timepoints is shown as a difference image.

Motion‐encoded radial k‐space data were created from image sequences by combining spokes of readout data from sequential “static” four times undersampled k‐space data generated with the NUFFT procedure described above (see Figure 2). AWGN at 25 dB was added to raw k‐space data to simulate sensor noise. For ground truth data, we selected the image corresponding to the last frame in each k‐space acquisition because knowledge of the most recent anatomical state is desired for real‐time beam adaptation on an MRI‐Linac.

Motion sequence data were augmented by a factor of 8 via a series of flips, rotations, and time‐reversal processes. Data were further augmented by the addition of random phase maps. For final model training, this YouTube‐8M dataset was combined with the ImageNet dataset described above. A separate motion test set of 1000 motion sequences and input data were created from an independent 218 videos in the YouTube‐8M dataset using the SSIM criteria described above.

As a testing tool, a time‐series of 2D lung cancer images were generated using the digital CT/MRI breathing XCAT (CoMBAT) phantom for a balanced steady‐state free precession (bSSFP) sequence with TR/TE = 10/5 ms.^29^, ^36^, ^37^ Image sequences were transformed to 4 × undersampled k‐space data using the process shown in Figure 2.

### 2.4 Image reconstruction

Neural network image reconstruction was performed by running inference on flattened radial input data with the corresponding, trained AUTOMAP model. The AUTOMAP reconstruction time was measured as wall time taken to perform this inference step in Keras on an unburdened workstation as measured over 20 repeats.

Conventional CS and NUFFT image reconstruction techniques were performed using the Berkeley Advanced Reconstruction Toolbox (BART).^38^ The NUFFT reconstruction interpolates k‐space data onto a Cartesian grid and then performs a fast Fourier transform.^39^ The CS implementation utilizes a NUFFT with an iterative algorithm to find the *l*1‐regularized solution to

(2) $$\min_{x}\left\{{∥Ax-y∥}_{2}+\lambda {∥\psi x∥}_{1}\right\},$$

where y is the acquired k‐space, A is the (under)sampling operator over the reconstructed image x, λ is a regularization parameter, and ψ is the wavelet operator. A grid search was used to optimize λ in the range $10^{-7}-10^{-1}$ for a minimum normalized root mean square error (NRMSE) with 30 iterations. Reconstruction times for CS are the self‐reported time for reconstruction as measured by the BART toolbox over 20 repeats.

The NRMSE was used as the primary metric to evaluate reconstruction quality and is calculated as the RMSE between reconstructed image and ground truth divided by the intensity range of the ground truth image. Structural similarity (SSIM) and the peak signal‐to‐noise ratio (PSNR) are also considered as additional quantitative metrics that may indicate the clinical utility of the images.^40^ In results quantifying the reconstruction quality for lung cancer patient images, the bar chart values reported for SSIM/NRMSE are the mean and standard deviation across 400 image slices in the two patient subset of lung cancer dataset that was unseen by AUTOMAP during training. We performed a paired *t*‐test to evaluate the significance of the difference between reconstruction metric distributions yielded from image slices in the testing dataset. Metric distributions were additionally evaluated with a Kolmogorov–Smirnov test to ensure that normality requirements for parametric statistical tests were satisfied.

### 2.5 Template matching

To simulate target tracking, regions of interest encompassing the tumor and diaphragm were defined in the first ground truth image of the digital phantom. Using CS and trained neural networks, stacks of 240 images were reconstructed from data retrospectively acquired with cardiothoracic motion. A template matching algorithm based on OpenCV software then calculated the closest matching target location from these reconstructed images with a normalized cross‐correlation algorithm at half‐pixel resolution (1 mm).^7^

### 2.6 Multichannel data processing and reconstruction

To explore the potential application of AUTOMAP to reconstruction of multichannel radial MRI data, we trained AUTOMAP to perform a four‐channel ($n_{c}=4$) reconstruction for *R* = 8. AUTOMAP training was performed using the same ImageNET and lung cancer patient datasets described in Section 2.1 for n=128. Four‐channel sensitivity maps for an idealized birdcage coil were defined using included functionality in SigPy.^41^ Four‐channel k‐space data were subsequently generated via a NUFFT operation after applying sensitivity maps to images. To simulate real‐world sensor noise, 25 dB of AWGN was applied to the two‐patient test dataset.

Multichannel reconstructions were performed using a conventional NUFFT root‐sum‐of‐squares approach (RSS) and CS for comparison to AUTOMAP. In the NUFFT RSS approach, each channel is reconstructed individually, and the result is the root‐sum‐of‐squares of images from all channels. To perform CS reconstruction, sensitivity maps were derived with the ESPIRiT tool in BART from multichannel images reconstructed at low (24× 24) resolution with NUFFT.^42^ Parallel imaging CS reconstruction was subsequently performed with the BART toolbox as described in Section 2.4 but with the additional use of the ESPIRiT‐derived sensitivity maps. The CS regularization parameter was optimized via a grid search for the $n_{c}=4$, R=8 multichannel reconstruction.

## 3 RESULTS

### 3.1 Model training

Models trained for static reconstruction on ImageNet derived data converged smoothly for all undersampling factors tested. The training time increased with model size (see Table 1 for the number of parameters in each model). The training time per epoch was in the range 5 min. (*R* = 16) to 15 min. (*R* = 1). A plateau in validation cost was found within 300 epochs of training for networks with *R* > 4. Models pretrained on ImageNET data were then fine‐tuned on a lung cancer images. For example, with *R* = 4, the fine‐tuned AUTOMAP model had a validation cost 9.7 times lower than a model trained from scratch on the lung cancer training data, emphasizing the value of transfer learning from generic pretrained models when only smaller datasets are available for a given anatomy. Examples of training/validation cost training dynamics and further analysis are included in Figure S2.

**TABLE 1:** Computational load of image reconstruction techniques. The time to perform single‐channel image reconstruction with AUTOMAP and compressed sensing techniques for different undersampling factors is shown in addition to the number of trainable parameters in AUTOMAP. Compressed sensing reconstructions utilize one optimized hyperparameter value for each undersampling factor.

**Acceleration factor (*R*)**		1	2	4	8	16
	AUTOMAP 1‐Slice	16.0 ± 0.3	10.4 ± 0.4	7.2 ± 0.6	5.3 ± 0.7	4.7 ± 0.9
**Reconstruction time (ms)**	AUTOMAP 10‐Slices	19.1 ± 0.4	13.5 ± 0.4	10.0 ± 0.4	8.2 ± 0.7	7.1 ± 0.8
	Compressed Sensing 1‐slice	253 ± 23	240 ± 17	235 ± 8	236 ± 10	231 ± 21
**Number of AUTOMAP parameters**		1.96B	1.12B	696M	487M	378M
**CS regularization parameter (** *λ*))		0.005	0.005	0.005	0.01	0.05

### 3.2 Neural network reconstruction performance

**FIGURE 3:**
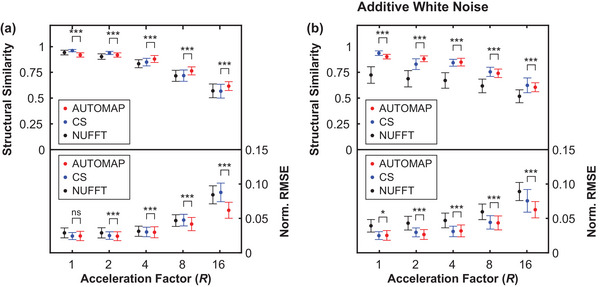
Quality of radial AUTOMAP reconstruction in comparison to conventional techniques. Reconstruction quality as measured via structural similarity and normalized root mean square error metrics (Norm. RMSE) for different undersampling factors. In general, a more accurate image reconstruction technique yields a high structural similarity value and a low normalized RMSE value. Results are shown for AUTOMAP (red), compressed sensing (CS, blue), and nonuniform fast Fourier transform (NUFFT, black). Resulting image quality was assessed for clean radial input data (shown in **a**) and for the same data with 25 dB of additive white Gaussian noise (shown in **b**). Data markers have been offset from the acceleration factor values shown to aid visual clarity. Error bars represent the standard deviation of metrics across the test dataset. *p < 0.05, **p < 0.01, ***p < 0.001, ns = no significant difference

**FIGURE 4:**
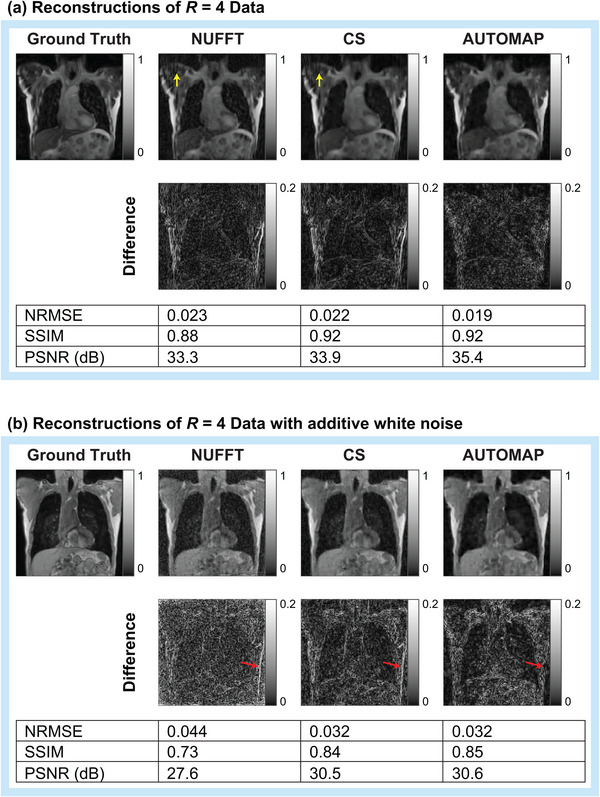
Visual comparison of AUTOMAP reconstruction performance to conventional techniques. AUTOMAP is compared to compressed sensing (CS) and nonuniform fast Fourier transform (NUFFT) techniques in images reconstructed from 4× undersampled golden‐angle radial data. Images from clean radial data (a) and from data with 25‐dB additive white Gaussian noise (b) are shown. Normalized root‐mean‐square error (NRMSE), structural similarity (SSIM), and peak signal‐to‐noise ratio (PSNR) metrics are shown. Yellow arrows in a indicate streaking artifacts (zooming on the electronic version may aid visibility). Red arrows in (b) are provided for discussion in the text.

With models trained for the neural network reconstruction task, we now present key results in Figures 3 and 4 that compare the reconstruction error of AUTOMAP to established methods.

In Figure 3, we show structural similarity (SSIM) and NRMSE metrics for images reconstructed from golden‐angle radial k‐space data derived from the lung cancer imaging test set at a range of acceleration factors (*R*). We note that the SSIM metric quantifies the structural quality of reconstructions in terms of changes in the inter‐dependency of pixels that are spatially close, while the NRMSE metric quantifies the absolute error in pixel intensities. In general, higher SSIM values and lower NRMSE values correspond to reconstructions that are perceived by radiologists to be of higher diagnostic quality.^40^ AUTOMAP performs strongest with very sparsely sampled data, giving an NRMSE value 0.70 times that of CS reconstruction for R=16 data as shown in Figure 3a. We observe that NUFFT reconstructs the “clean” data effectively, having an NRMSE value that is only 1.04 times higher than the NRMSE value for CS when R=4 (Figure 3a). However, when white noise, representing the thermal noise present in a standard MRI experiment,^43^ is added to the input k‐space data, we observe considerable deterioration in the accuracy of NUFFT reconstructions performed with the NRMSE being 1.52 times higher than for CS at R=4 (Figure 3b). For fully sampled data with white noise added (R=1), CS and AUTOMAP have mean NRMSE values of 0.0245 and 0.0247, indicating small differences in reconstruction performance that are on the threshold of statistical significance (*p* = 0.046).

Aware that NRMSE and SSIM metrics do not fully characterize artifacts that may be present in reconstructed images, we present typical reconstructions for *R* = 4 shown in Figure 4. While, the overall quality trend is similar to that summarized in Figure 3, we observe that the AUTOMAP difference image shows significantly lower error at the chest wall than CS and NUFFT techniques (see red arrows in Figure 4b). We also note that subtle streaking artifacts are present in the CS and NUFFT images but absent from AUTOMAP reconstructions (see yellow arrows in Figure 4a). Increasing the regularization penalty was observed to improve the structure of CS reconstructed images at the expense of a poorer NRMSE (see Figure S3 for results showing the trade‐off between NRMSE and SSIM when optimizing the regularization penalty).

Having shown that AUTOMAP reconstructs input data with equivalent or superior fidelity to CS, we now evaluate the relative computational performance of the reconstruction methods. We found that our implementation of AUTOMAP was 16–49 times faster than CS reconstruction, reducing reconstruction time by approximately 200 ms (see Table 1). For context, the end‐to‐end imaging and targeting latency should be less than ∼500 ms for real‐time tumor tracking in MR‐guided radiotherapy.^5^ The speed of NUFFT reconstruction was not quantified, as it is accepted that this less robust reconstruction technique can be completed within several milliseconds, making it sufficiently fast for real‐time applications.^44^ We found that CS reconstruction was fastest running on the CPU without GPU acceleration, presumably due to the relatively small matrix sizes used, and hence, report the CPU‐based times here.

The number of trainable parameters required to implement AUTOMAP were significant (Table 1), reaching up to 2B for the fully sampled network. A single *l*1‐penalty hyperparameter (λ) was optimized for CS recon.

### 3.3 Motion‐compensated reconstruction

Here, we test the potential of the AUTOMAP reconstruction technique to correct for anatomical motion encountered during the acquisition process, which is of concern for MRI‐guided RT of thoracic sites in particular. In this section, we consider two AUTOMAP models trained to reconstruct data acquired with *R* = 4. The first AUTOMAP model is the same as the *R* = 4 model trained for reconstruction as described above but without fine‐tuning. The second AUTOMAP model was trained to compensate for intra‐acquisition motion through the incorporation of generic motion data from YouTube‐8M into the training dataset.

**FIGURE 5:**
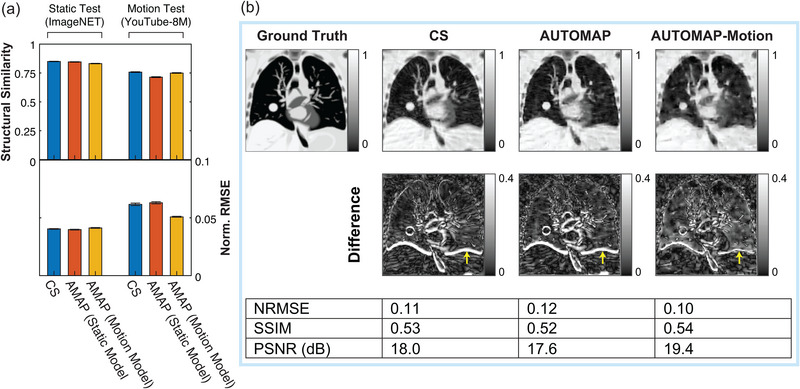
Reconstructing motion‐corrupted, undersampled data. (a) Reconstruction quality as measured via structural similarity and normalized root mean square error metrics (Norm. RMSE) for static test images derived from the ImageNET database and for motion‐corrupted test inputs derived from the YouTube 8M database. Results are shown for 4× undersampled data reconstructed with compressed sensing (blue), an AUTOMAP model trained on static data (red) and an AUTOMAP model trained on motion‐encoded data. Bars and lines are the mean and standard error of the mean calculated across 1000 test inputs. (b) Images reconstructed from k‐space data simulated with the CoMBAT phantom for a patient under routine cardiothoracic motion. Results for data reconstructed with compressed sensing, an AUTOMAP model trained on static data, and an AUTOMAP model trained on motion‐encoded data are shown. Normalized root‐mean‐square error (NRMSE), structural similarity (SSIM), and peak signal‐to‐noise ratio (PSNR) image quality metrics are evaluated against the last frame of the image sequence for motion‐encoded data. Yellow arrows indicate errors associated with the position of the diaphragm in reconstructed images.

We begin analyzing these models by comparing the quality of CS reconstruction to the AUTOMAP model reconstructions for static and motion inputs in Figure 5a. The quality of all reconstructions is higher for static test data than motion test data, which reflects the increased difficulty of reconstructing motion‐corrupted data. We note that due to the large test sets that could be derived from ImageNET and YouTube‐8M, differences between all metrics summarized in Figure 5a are statistically significant at the *p*<0.005 level. The best performing technique on motion test data was the motion‐trained AUTOMAP model, which had a mean NRMSE 21% lower than the CS reconstructions. Additionally, the performance of AUTOMAP was comparable on static input data whether or not the model was trained with the inclusion of motion data, indicating that motion training leverages previously underutilized capacity in the over‐parameterized model architecture. Conversely, we note that AUTOMAP trained on the YouTube‐8M dataset alone had a minimum validation loss 68% higher than when static data were included in the training set, likely due to the difficulty of fitting to the underlying manifold with variability in the motion‐encoded data.

Having evaluated the performance of these reconstruction techniques on generic motion sequences, we now turn to analyze the performance of these models in reconstructing motion‐corrupted k‐space data simulated from the CoMBAT phantom for a lung cancer patient undergoing realistic cardiothoracic motion.^45^ The reconstruction results for CoMBAT data encoded as per the process described in Figure 2 are shown in Figure 5b. AWGN at 25 dB was added to all inputs used to derive this figure. Given that we are reconstructing motion‐corrupted data, the quantitative metrics of reconstruction performance are consistent with the results in Figure 5a, with the motion‐trained AUTOMAP model outperforming the static‐trained model and CS. Inspecting difference images, we see that the output of the motion‐trained AUTOMAP model has the least discrepancy with the ground truth around the diaphragm as indicated by yellow arrows in Figure 5b.

**FIGURE 6:**
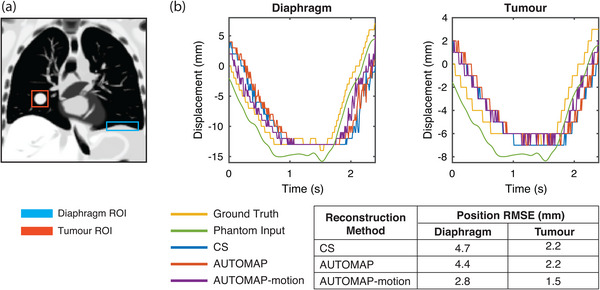
Target tracking accuracy. (a) Regions of interest (ROIs) for the diaphragm (blue) and tumor (red) are defined in a ground truth image. (b) Displacement of ROIs defined in a as predicted by a template matching algorithm for the ground truth image sequence (yellow) and image sequences reconstructed using compressed sensing (CS, blue), a conventional AUTOMAP model (red) and an AUTOMAP‐motion model (purple). Steps in displacement reflect the underlying image resolution. Root mean square error values are calculated for the difference between target position in reconstructed image sequences and the ground truth image sequence. The motion trace input to the virtual phantom is shown (green) with a vertical offset for visibility.

To simulate the impact of motion‐training on an MRI‐Linac tracking experiment, we performed template matching of tumor and diaphragm ROIs in our digital phantom, as shown in Figure 6. Analyzing the difference between template match locations in ground truth images and reconstructed images through one respiratory cycle, we find that the AUTOMAP‐motion model gives an RMSE value 1.9 mm smaller for the diaphragm and 0.7 mm smaller for the tumor than CS.

### 3.4 Multichannel reconstruction

**FIGURE 7:**
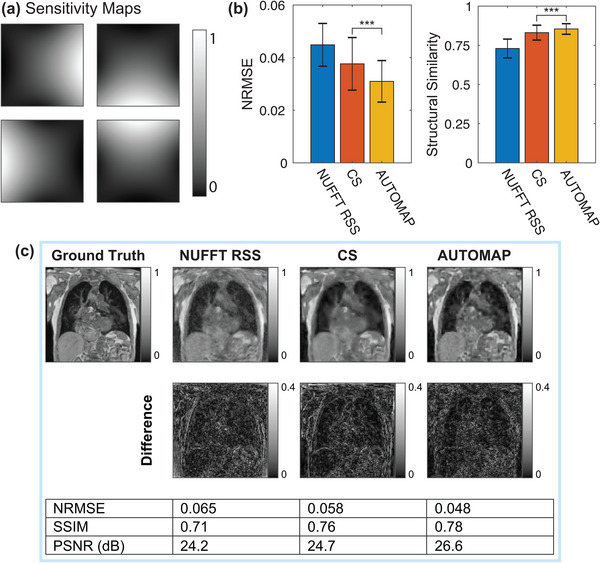
Multichannel reconstruction performance of nonuniform fast‐Fourier transform root‐sum‐of‐squares (NUFFT RSS), Compressed sensing (CS) and AUTOMAP techniques. (a) Sensitivity maps used for four‐channel data acquisition and reconstruction with a reduction factor (*R*) of 8. (b) Average normalized root‐mean‐square error (NRMSE) and structural similarity metrics for each technique as measured across the test set (error bars denote standard deviation). (c) Visual comparison of reconstruction performance. NRMSE, structural similarity (SSIM), and peak signal‐to‐noise ratio (PSNR) metrics are shown. Error bars represent the standard deviation of metrics across the test dataset. *p < 0.05, **p < 0.01, ***p < 0.001, ns = no significant difference

Multichannel reconstructions were performed with data acquired using sensitivity maps for a four‐channel birdcage coil (maps shown in Figure 7a). Metrics quantifying the performance of AUTOMAP, NUFFT RSS, and CS reconstruction approaches are shown in Figure 7b. The multichannel results show that AUTOMAP was superior to NUFFT RSS and CS reconstruction approaches in terms of structural similarity and NRMSE metrics for the $n_{c}=4$, R=8 case explored here. A visual comparison of multichannel reconstruction performance is shown in Figure 7c.

## 4 DISCUSSION

Our results leverage advances in machine learning to implement fast image reconstruction of undersampled radial data from lung cancer patients with comparable accuracy to conventional iterative reconstruction techniques based on CS. The development of rapid radial reconstruction techniques is of interest for MRIgRT, where the latencies associated with iterative reconstruction are prohibitively long.^46^ While our proof‐of‐principle study has focused on the application of AUTOMAP to real‐time targeting of radiotherapy in the lung, we believe our results are extensible to high‐motion sites such as the liver and prostate, where tumor movement would be optimally managed by real‐time adaptive radiotherapy.^47^, ^48^ We note that due to the relatively high latency of MR acquisition and reconstruction, compared to X‐ray‐based modalities, faster image reconstruction techniques are desired for real‐time beam gating and MLC tracking on MRI‐Linacs, especially for non‐Cartesian acquisition trajectories.^7^, ^49^ Integrating neural networks with fast data streaming tools,^50^ tracking algorithms,^51^ and time‐resolved 3D anatomical imaging^52^, ^53^ will be crucial for use with MRI‐Linac beam adaptation technologies.

The ease with which AUTOMAP generalizes to different reconstruction tasks, like the golden‐angle radial sampling used in this work, is a direct consequence of the sequential dense layers in the model architecture. While these dense layers enable data‐driven learning of the manifold between k‐space and the image domain, they also have significant memory requirements that make the translation to 3D reconstruction and tracking more challenging.^54^, ^55^ To perform reconstructions above the relatively low resolutions used for motion tracking on an MRI‐Linac will require lighter‐weight reconstruction networks.^17^, ^56^ One light‐weight implementation of AUTOMAP is decomposed‐AUTOMAP (dAUTOMAP), which replaces dense layers with orthogonal “domain transform” layers.^20^ While dAUTOMAP performs strongly for Cartesian trajectories, it assumes that data are acquired in orthogonal directions, making it unsuitable for reconstruction of nonuniform data. Hence, dAUTOMAP is outperformed by NUFFT reconstruction for radial reconstructions.^23^ We note that light‐weight architectures that take view angle into account are an active research area.^25^, ^57^

Without a detailed understanding of the AUTOMAP learning process, it may also seem surprising that the neural network trains faster and performs better relative to CS at high acceleration factors (see Figure 3). However, this reflects the underlying mathematics of AUTOMAP as a tool for manifold approximation, where image reconstruction is achieved via a mapping between sparse representations in signal and image space. Hence, highly undersampled data encourages the network to learn robust, low‐dimensional, latent representation of data that can then be used for manifold approximation.^58^ Future work will aim to improve network training via the use of loss metrics other than MSE and via the imposition of layer regularization penalties that promote sparsity. In the present work, we have chosen to focus on golden‐angle radial sampling trajectories as they are relatively insensitive to motion artifacts, which is a significant benefit for applications in MRI‐guided radiotherapy.^2^ However, we note that future opportunities exist to investigate how reconstruction accuracy is impacted by increasing acceleration factors for data acquired with spiral and cartesian sampling trajectories.

Our experiments with motion‐encoded k‐space demonstrated that as a highly over‐parameterized model, AUTOMAP has significant capacity to learn additional features. Here, we showed that AUTOMAP could learn generic properties of motion from YouTube videos, leading to lower NRMSE and higher tracking accuracy in reconstructed images of an *in silico* lung cancer phantom. The relatively low quality of images reconstructed with conventional techniques from this undersampled, motion‐corrupted data indicates the significant potential impact of improved reconstruction frameworks based on machine learning. Further, our tracking results do not account for reconstruction latency, which would be expected to further reduce tracking accuracy with CS. Additionally, there is a significant scope to improve on tracking and reconstruction performance through utilization of more in‐domain training data such as medical dynamic MRI data. However, care must be taken when using dynamic MRI data during the supervised learning process as the ground truth is generally unknown due to intra‐acquisition motion. One alternative is to train networks with synthetically generated motion applied to lung images. Such an approach could be adapted to assist with tracking of targets with out‐of‐plane motion, which represents a persistent challenge in MRI‐guided radiotherapy.^59^ Temporal k‐space filtering,^9^ optical flow techniques,^23^ and neural networks tailored to radial reconstruction could also improve network performance.^57^, ^60^, ^61^

Our results show that AUTOMAP performs well with the addition of white noise, which represents the fundamental thermal limitations to SNR in an MRI scan. However, another valuable strength of manifold‐based reconstruction models, such as AUTOMAP, is that they implicitly learn to suppress MRI artifacts that are caused by inputs outside the training domain, for example, spike noise resulting from RF leakage.^55^

Retrospective simulation of k‐space data from DICOMs, as performed in our experiments, can lead to overly optimistic reconstruction results and future work with raw k‐space data will be required to test the robustness of AUTOMAP to other experimental imperfections encountered in MRI, such as *B*_0_ and *B*_1_ inhomogeneity.^62^ While the over‐parameterization of AUTOMAP means that it can be intentionally trained to correct for such artifacts, the incorporation of such specific examples into the training corpus will increase the risk of overfitting. Despite the risk of overfitting, it is likely that performance of AUTOMAP will benefit from some fine‐tuning of pretrained networks to the particular MRI system being used. The incorporation of new adversarial approaches into the training corpus will make neural network reconstructions more robust by identifying nonphysical input perturbations that can negatively impact reconstruction performance.^63^, ^64^

In this work, we have focused on CS with l1‐wavelet regularization as it is widely used and well‐supported in the MRI community. However, other effective regularization methods (e.g., locally low rank) remain to be tested. We note that all standard iterative reconstruction techniques will be slow when compared to frameworks such as AUTOMAP that can reconstruct data in a single forward pass.

We demonstrated that AUTOMAP can be extended to parallel imaging reconstruction tasks based on multicoil acquisitions. However, we note that the sensitivity maps used in our experiment were fixed and further work will be required to ensure that AUTOMAP performs well with variations in sensitivity maps between patients. Future work will be required to extend the reconstruction framework to generalized sensitivity maps that are encountered clinically.^65^

In conclusion, we have used AUTOMAP to accurately and rapidly reconstruct retrospectively acquired lung cancer images from radial data. We have also shown that AUTOMAP can adapt to generalized properties of motion learned from generic YouTube videos for real‐time tracking applications. These results will inform the future development of dynamic adaptation technologies for MRI‐Linacs, enabling new standards of personalized radiotherapy.

## CONFLICT OF INTEREST

P.J.K. is an inventor on two patents relating to MRI‐Linac systems: US#8,331,531 and US#9,099,271. M.S.R. and N.K. have received research support from GE Healthcare for MR image reconstruction projects. The remaining authors have no relevant conflicts of interest to disclose.

## Supporting information


Supporting Information

## Data Availability

The primary datasets used for training in this study are publicly available. Code for preprocessing data and model training is available at https://github.com/MattRosenLab.
