# Supplementary material for: Real‐time radial reconstruction with domain transform manifold learning for MRI‐guided radiotherapy
Source: Med Phys. 2023 Jan 27;50(4):1962–74. doi: 10.1002/mp.16224 (PMC10809819; doi:10.1002/mp.16224)
Supplement: Supplementary file 1 — Supporting Information [file MP-50-1962-s001.pdf]

Supplementary Material: Real-time Radial Reconstruction with Domain Transform Manifold Learning for MRI-guided Radiotherapy

David E. J. Waddington,[1,2,3,*] Nicholas Hindley,[1,3] Neha Koonjoo,[3]
Christopher Chiu,[1] Tess Reynolds,[1] Paul Z. Y. Liu,[1,2] Bo Zhu,[3] Danyal Bhutto,[3,4] Chiara Paganelli,[5] Paul J. Keall*,[1,2] and Matthew S. Rosen*[3,6,7]

[1]*ACRF Image X Institute, Faculty of Medicine and Health, The University of Sydney, Sydney, Australia*

[2]*Department of Medical Physics, Ingham Institute for Applied Medical Research, Liverpool NSW, Australia*

[3]*A. A. Martinos Center for Biomedical Imaging, Massachusetts General Hospital, Charlestown, MA, USA*

[4]*Department of Biomedical Engineering, Boston University, Boston, MA, USA*

[5]*Dipartimento di Elettronica, Informazione e Bioingegneria, Politecnico di Milano, Milan, Italy*

[6]*Department of Physics, Harvard University, Cambridge, MA, USA*

[7]*Harvard Medical School, 25 Shattuck St., Boston, MA, USA*

[*] david.waddington@sydney.edu.au

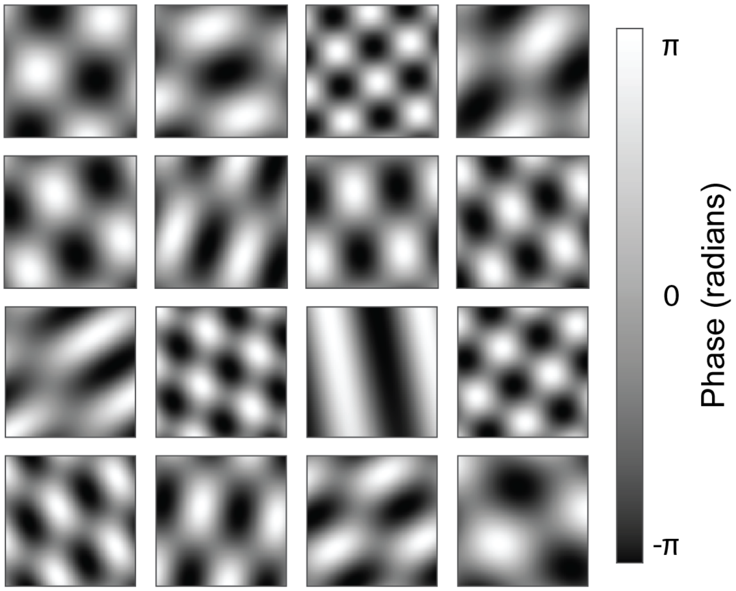

Supplementary Figure 1. **Examples of synthetics phase maps.** Image data were augmented with synthetic phase maps generated from two-dimensional sinusoidal waves with randomly selected frequency and phase offsets.

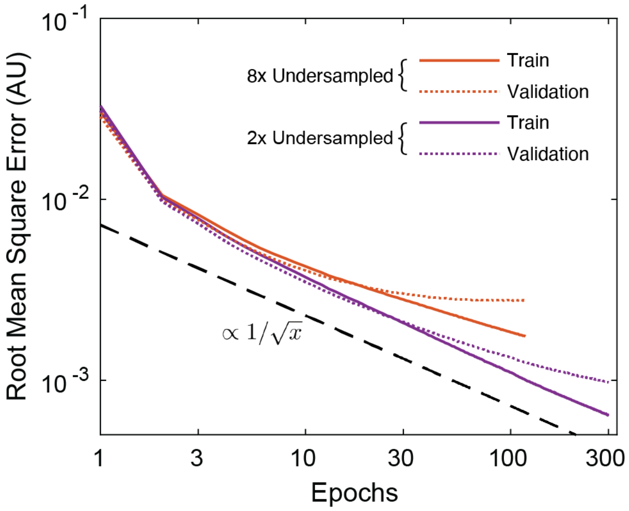

Supplementary Figure 2. **Training AUTOMAP to reconstruct radial MRI data.** Examples showing the convergence dynamics of training set costs (solid lines) and validation set costs (dotted lines) as AUTOMAP is trained to reconstruct radially-sampled data .at two different undersampling factors. Data for acquisition trajectories undersampled by a factor of 2 (purple) and 8 (red) in the phase-encode direction are shown. The dashed line proportional to the inverse square of epoch number (x) is included as a guide to the eye and for discussion in the text. We note that while long, the convergence time for networks with $R = 1{,}2$ is finite, with the training cost measured by RMSE reducing as roughly the inverse square root of the number of epochs. Models were found to fit to training data successfully with batch sizes in the range 5-100, with fluctuations in the minimum validation costs reached being less than $\sim$10%. The training behaviors observed are consistent with general properties of over-parameterized networks, such as AUTOMAP, where poor local minima do not play a significant role in the training dynamics which are instead governed by a high number of flat directions in the cost landscape, allowing training to be achieved.[1, 2] Regularization methods to improve the fit performance of AUTOMAP to training/validation data were trialed, including dropout and the addition of multiplicative noise but resulted in poorer validation costs, further indicating that AUTOMAP is fitting well to the underlying reconstruction model.

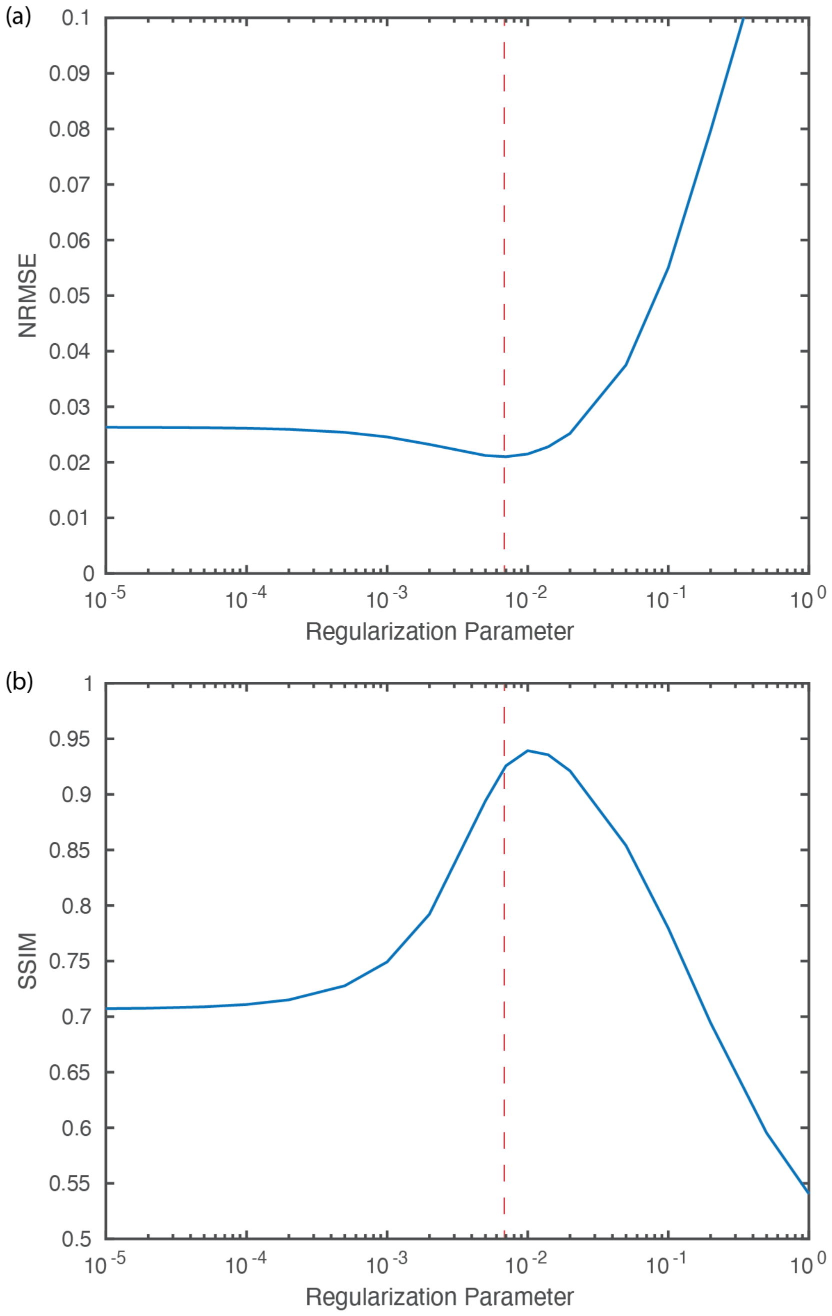

Supplementary Figure 3. **Optimizing regularization parameters for compressed sensing reconstruction.** **(a)** The normalized root mean square error (NRMSE) of a 4$\times$ undersampled image reconstructed with different values of the regularization parameter (λ). The regularization parameter corresponding to a minimum in NRMSE was selected for testing via a grid search on training data for each undersampling factor. **(b)** The structural similarity (SSIM) of a 4$\times$ undersampled image reconstructed with different values of the regularization parameter (λ). The red dotted line indicates the regularization parameter corresponding to a minimum in NRMSE. Noticeably, the minimum in NRMSE and the maximum in SSIM metrics, which correspond to the highest quality reconstructions, occur at different regularization values.

Supplementary References

[1] Z. Allen-Zhu, Y. Li, and Z. Song, arXiv , 1811.03962 (2019), arXiv:1811.03962.

[2] M. Baity-Jesi, L. Sagun, M. Geiger, S. Spigler, G. Ben Arous, C. Cammarota, Y. Lecun, M. Wyart, and G. Biroli, Journal of Statistical Mechanics: Theory and Experiment , 124013 (2019), arXiv:1803.06969.
